# Atomic-Scale Insights into the Effects of the Foaming Degree on the Glass–Ceramic Matrix Derived from Waste Glass and Incineration Bottom Ash

**DOI:** 10.3390/ma17122820

**Published:** 2024-06-10

**Authors:** Ying Wei, Ziwei Chen, Hao Wang

**Affiliations:** 1 College of Civil Engineering and Architecture, Zhejiang University, Hangzhou 310030, China; 19121127@bjtu.edu.cn; 2 Department of Civil and Environmental Engineering, The Hong Kong Polytechnic University, Hung Hom, Kowloon, Hong Kong 999077, China; 3 Research Centre for Resources Engineering towards Carbon Neutrality, The Hong Kong Polytechnic University, Hung Hom, Kowloon, Hong Kong 999077, China; 4 School of Energy and Environmental Engineering, University of Science & Technology Beijing, Beijing 100083, China; wanghao@ustb.edu.cn

**Keywords:** atomic structure, MD simulations, foaming glass–ceramics, total porosity, compressive strength, oxygen species

## Abstract

Precise management of the inverse correlation between the total porosity and compressive strength is crucial for the progress of foaming glass–ceramics (FGCs). To deeply understand this relationship, we investigated the atomic-level transformations of five CO_2_-foaming FGC samples using molecular dynamics simulation. The short-range and intermediate-range structures of the FGCs with varying total porosities (36.68%, 66.28%, 66.96%, 72.21%, and 79.88%) in the system were elucidated. Na cations were observed to exhibit a strong interaction with CO_2_, accumulating at the surface of the pore wall and influencing the oxygen species. Therefore, the change in the atomic structure of the matrix was accompanied by an increase in the total porosity with an increasing CO_2_ content. Specifically, as the total porosity increased, the bridging oxygen content within the FGCs rose accordingly. However, once the total porosity exceeded 66.96%, the bridging oxygen content began to decline. This observation was significant considering the role of the bridging oxygen content in forming a continuous cross-linked network of chemical bonds, which contributed to the enhanced mechanical strength. Consequently, the influence of the total porosity on the oxygen species resulted in a two-stage reduction in the compressive strength. This study offers valuable insights for the development of high-strength lightweight FGCs.

## 1. Introduction

Foaming glass–ceramics (FGCs), distinguished by their distinctive three-dimensional spatial mesh structure, are widely recognized for their high porosity. The presence of pores substantially enhances the exceptional acoustic and thermal insulation properties of FGCs, facilitating their utilization in various industrial and construction applications [1,2,3]. As one of the main technologies to produce FGCs, the powder sintering method initially comprises of blanks wrapped around the foaming agent to form a compact block. As the temperature rises during firing, a liquid phase forms around the foaming agent. Subsequently, the gas generated by the foaming agent becomes encapsulated by the high-temperature liquid phase, initiating the formation of pores with varying sizes. Over time, these pores expand and coalesce, culminating in the distinctive three-dimensional spatial mesh structure characteristic of FGCs. Therefore, it is evident that the properties of the raw materials play a crucial role in the production of FGCs.

Compared with natural silicate materials like clay and quartz, utilizing solid waste as the main source of raw materials for high-strength FGCs enhances the environmental value, reduces preparation costs, and promotes sustainable development [4]. Yio et al. [5] produced FGCs from coal-fired power station furnace bottom ash and soda–lime–silica glass. Wang et al. [6] utilized the high-temperature decomposition properties of desulfurization gypsum as a foaming agent, successfully preparing foam glass–ceramics from fly ash, blast furnace slag, and desulfurization gypsum. Liu et al. [7] obtained green FGCs with the lowest bulk density (0.63 g/cm³) and a suitable mechanical strength (9.7 MPa) by adjusting the silica sand tailings content. According to our previous work [8], FGCs derived from incineration bottom ash, waste glass, and waste paper were successfully designed and prepared. The foaming mechanism, the cellulose pyrolysis of waste paper, produces reducing substances under the anoxic interior environment of the FGC. The reaction of the reducing substances with Fe_2_O_3_ in the incineration bottom ash generates CO_2_ gas, leading to the foaming of the FGC sample. The compressive strength of the FGCs was found to be positively correlated to its geometric density, a trend consistent with findings from other studies [9,10]. Porosity, a property intricately linked to geometric density, has been investigated by numerous researchers, resulting in the proposal of various models. A simple equation, initially proposed by Ryshkewitch [11] and later refined by Rice [12,13], has been widely used to analyze the relationship between the strength and porosity. This model can be approximated as follows:

(1) $$\sigma =\sigma_{0}\exp(bp)$$

where σ and $\sigma_{0}$ are the strength of a material with the total porosity p and the strength of the same material without porosity, respectively, and b is a constant dependent on the pore characteristics.

**Figure 1:**
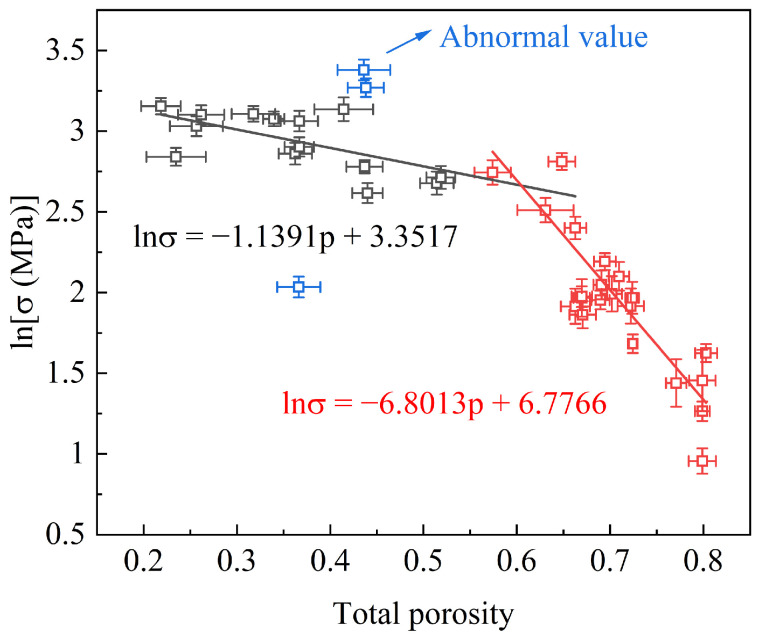
Compression strength vs. total porosity for FGCs.

A collection of data for the compressive strength as a function of the total porosity (TP) for the FGC samples in our previous study [8] is depicted in Figure 1. Regression analysis was employed using the Ryshkewitch–Rice model to investigate the relationship between the strength and TP. Initially, the mechanical strength exhibits a gradual decline with the increasing TP. However, beyond a critical TP threshold, a notable acceleration in the rate of the strength reduction was observed. This departure from previous research findings underscores the intrinsic relationship between the strength and TP. Furthermore, the observed two-stage reduction in the strength suggests that the turning point harbors the potential for attaining a harmonized equilibrium between the strength and TP.

To elucidate the intricate relationship, molecular dynamics simulation was employed to investigate the atomic-scale effects of the TP on the FGCs. The simulations comprehensively captured both short- and intermediate-range structural information. Specifically, short-range structural features were delineated through the pair distribution function (PDF) and coordination number (CN) analysis of cation–oxygen pairs. Intermediate-range structures, encompassing Al- and Si-related units, along with equilibrium constants of Q^n^(Si) species and oxygen species, were quantitatively computed and validated against Fourier transform infrared (FTIR) results. This microscopic information was utilized to elucidate the observed two-stage changes in macroscopic strength experiments. Furthermore, this analysis presents a fresh perspective on optimizing the material efficiency in the manufacturing of foamed ceramics with both a high TP and high strength.

## 2. Materials and Methods

### 2.1. Experimental Design

The materials utilized in this study were sourced from local recycling facilities. Waste glass was obtained from crushed glass bottles and sheet glass, incineration bottom ash was collected from the incineration plant, and waste paper was sourced from discarded printing paper found in office environments. The chemical compositions and phase compositions of the waste glass and incineration bottom ash were measured by X-ray fluorescence and are listed in Table 1. To explore the transformation of the relationship between the strength and TP, five sets of samples were selected for the mechanism analysis, namely, samples with 0 wt.% waste paper and a 5 min holding time, 15 wt.% waste paper and a 5 min holding time, 10 wt.% waste paper and a 5 min holding time, 12.5 wt.% waste paper and a 5 min holding time, and 20 wt.% waste paper and a 10 min holding time. Firstly, the waste glass and incineration bottom ash were crushed using a planetary ball milling machine, and the waste paper was cut with a paper shredder. The raw materials were mixed evenly with 0–20 wt.% waste paper using a mixer according to the designed ratio. Subsequently, the dried materials were shaped into spherical pellets using a disc granulator, with approximately 40 wt.% water added. These green samples, with a diameter of 15 mm, were then dried further in a laboratory oven at 105 °C for 24 h. Following the drying process, the samples were subjected to heat treatment in a muffle furnace. They were initially heated to 500 °C at a rate of 50 °C/min, and after holding at this temperature for 5 min, they were further heated to a peak temperature of 850 °C at a rate of 10 °C/min. The samples were then held at the peak temperature for 5 or 10 min before being cooled to room temperature within the muffle furnace.

The micro-level analysis of the FGC matrix was performed through Fourier transform infrared (FTIR) spectra and scanning electron microscope–energy dispersive spectroscopy (SEM-EDS, Tescan VEGA3, Brno, The Czech Republic). For the FTIR measurements, a 2.0 mg sample was blended with 200 mg KBr and compacted into a disk. Spectral data were collected using a spectrometer (SpectrumTwo, PerkinElmer, Waltham, MA, USA) across the range of 400 to 1300 cm^−1^ with a resolution of 2 cm^−1^.

### 2.2. All-Atomistic Molecular Dynamics Simulations

All-atomistic molecular dynamics (AAMD) simulations were conducted to investigate the foaming effects on the glass–ceramic matrix in a time-evolutional manner. The local structure of the above five groups of the FGC samples with different foaming degrees, corresponding to the total porosities (TPs) of 36.68%, 66.28%, 66.96%, 72.21%, and 79.88%, were simulated. The local compositions were in the 12.25 wt.% CaO–64.46 wt.% SiO_2_–2.42 wt.% Al_2_O_3_–3.91 wt.% FeO–14.48 wt.% Na_2_O system obtained from the thermodynamic equilibrium calculations by the FactSage 7.2 (Thermfact/CRCT, Canada and GTT-Technologies, Achen, Germany). The used potential fields and forcefield parameters are described as follows.

In the molten glass, the atomic dynamics are primarily determined by the two-body BMH potential [14,15], denoted as the pair potential $U_{ij}\left(r_{ij}\right)$, which describes the interaction between atoms i and j as a function of their separation distance $r_{ij}$. The $U_{ij}\left(r_{ij}\right)$ can be described as follows:

(2) $$U_{ij}\left(r_{ij}\right)=\frac{e^{2}q_{i}q_{j}}{4\pi \varepsilon_{0}r_{ij}}+A_{ij}exp\left(\frac{-r_{ij}}{B_{ij}}\right)-\frac{C_{ij}}{{r_{ij}}^{6}}+\frac{D_{ij}}{{r_{ij}}^{12}}$$

where q represents the effective charge associated with each atom.

In the simulation, the effective charge attributed to the oxygen atom is −0.945, while the charges assigned to other atomic species are determined via a prescribed charge allocation methodology, as delineated in previous works [16,17]. $\varepsilon_{0}$ denotes the dielectric constant, and $A_{ij}$, $B_{ij}$, $C_{ij}$, and $D_{ij}$ represent force constants governing interactions between atom pairs. The four components of this potential field encompass the long-range Coulombic interaction, short-range Born repulsion interactions, the van der Waals term, and an unphysical attraction occurring at distances of less than 1 Å. These terms collectively govern the interactions within the system. Specifically, the electrostatic interactions and short-range interactions between carbon and oxygen atoms (C and O*, respectively) encapsulate the interactions between CO_2_ molecules. The notation O* represents the oxygen atom within the CO_2_ molecules, distinct from the oxygen atom (O) present in the melt. The subsequent expression provides the potential energy $U\left(1,2\right)$ governing the interaction between CO_2_ molecules 1 and 2, expressed as follows:

(3) $$U\left(1,2\right)=\sum_{i\varepsilon 1}\sum_{j\varepsilon 1}\left[4\varepsilon_{ij}\left[\left[{\left(\sigma_{ij}/r_{ij}\right)}^{12}-{\left(\sigma_{ij}/r_{ij}\right)}^{6}\right]+\frac{e^{2}q_{i}q_{j}}{4\pi \varepsilon_{0}r_{ij}}\right]\right]$$

where $q_{C}$ and $q_{O^{*}}$ denote the effective charges located on the C and O* atoms with values of 0.5888 and −0.2944, respectively. Additionally, $\sigma_{ij}$ and $r_{ij}$ represent the Lennard–Jones (LJ) potential parameters governing the interactions between the corresponding pairs of C-C, C-O*, and O*-O* atoms.

The intramolecular potential, which adopts a harmonic form to accommodate molecular flexibility, is employed to accurately reproduce the bending vibration of CO_2_. This potential is described in detail in reference [18], as follows:

(4) $$U_{\theta }=1/2k_{\theta }{\left(\theta -\pi \right)}^{2}$$

where $k_{\theta }$ and θ denote the fixed force parameter and the bending angle, respectively.

To characterize the interactions between a CO_2_ molecule and the cation ($X_{j}$) within the melt, the potential energy is described by the following expression, as provided in reference [18]:

(5) $$U_{CO_{2}-X_{j}}=\sum_{i\varepsilon \left[{CO}_{2}\right]}\left[\frac{e^{2}q_{i}q_{j}}{4\pi \varepsilon_{0}r_{ij}}+A_{ij}exp\left(\frac{-r_{ij}}{B_{ij}}\right)-\frac{C_{ij}}{{r_{ij}}^{6}}+\frac{D_{ij}}{{r_{ij}}^{12}}\right]$$

where i represents the three atoms of CO_2_ molecule and j covers all cations in melt.

Furthermore, the interactions involving the C and the O within the melt are determined by the More potential, as described in reference [18], as follows:

(6) $$U_{C-O}=D_{e}\left[{\left(1-e^{-\left(r-l\right)/\lambda }\right)}^{2}-1\right]\;$$

where $D_{e}$ and l are the dissociation energy and the equilibrium distance of carbon–oxygen (C-O) bond, respectively. λ denotes the effective width of the potential.

All force field parameters mentioned were derived from extensively validated data reported in prior publications and are readily accessible in our preceding article.

**Figure 2:**
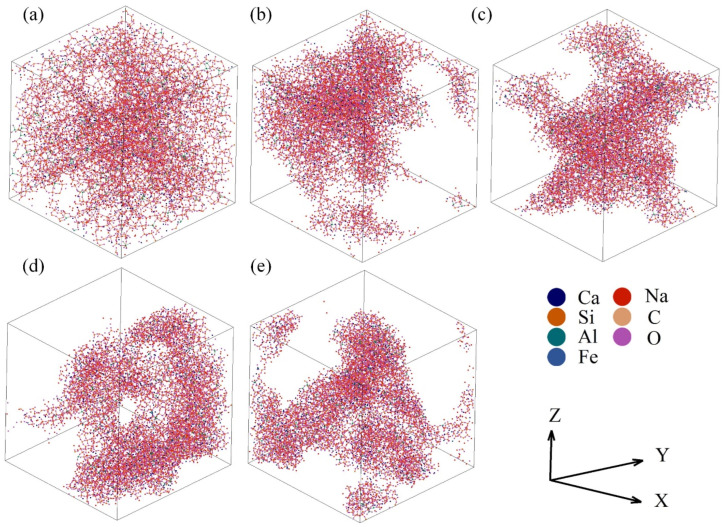
Structural configurations in the last picosecond of the samples with the total porosities of 36.68% (**a**), 66.28% (**b**), 66.96% (**c**), 72.21% (**d**), and 79.88% (**e**).

All simulations were finished using the massively parallelized open-source codes LAMMPS. The potential parameters used in the AAMD simulations are listed in Table 2. In the typical simulations, a cubic cell containing a total of ~10,000 atoms was created under periodic boundary conditions. All steps adopted the ensemble of constant temperature and volume and the Ewald summation method was used to calculate the long-range Coulombic energies with an 11 Å cut-off. The atomic configuration and box size were determined by the chemical composition and density of the sample. The initial structure arising from random generation was firstly thermalized at 5000 K for 200 ps and then quenched to the sintering temperature at a cooling rate of ~7.75 × 10^13^ °C/s and held for 100 ps. Next, the sample was slowly cooled down to 25 °C and continued to run for 200 ps to reach equilibrium. Figure 2 shows the last structural configurations of the samples with different total porosities. The last 100 ps of data were collected for average and statistical analysis.

### 2.3. Analysis Methods

The PDF, which characterizes the spherically averaged local organization surrounding a specific atom, was computed to encapsulate short-range structural information. The PDF equation is defined as follows [19]:

(7) $$g_{ij}\left(r\right)=\frac{V}{N_{i}N_{j}}\sum_{j}\frac{\langle n_{ij}\left(r-\frac{∆r}{2},r+\frac{∆r}{2}\right)\rangle }{4\pi r^{2}∆r}$$

where $N_{i(j)}$ represents the total number of atoms of type i(j), and for atoms of the same species, $N_{i(j)}=N_{i}-1$; V denotes the volume of the simulation box; $n_{ij}\left(r-\frac{∆r}{2},r+\frac{∆r}{2}\right)$ signifies the average number of atoms j surrounding atom i within the distance $\left(r-\frac{∆r}{2},r+\frac{∆r}{2}\right)$.

For each atomic pair, the initial peak observed in the respective PDF represents the distribution of distances within the first-neighbor shell. Specifically, in the case of the T-O pair, a subsequent minimum following this initial peak, termed the first valley of the PDF curve, serves as a robust indicator of the upper limit of this distribution. This upper limit, known as the cut-off distance, is pivotal in determining both the T-O coordination number and the fraction of the TOn polyhedral structures. The average CN can be derived by integrating the corresponding PDF curve up to the cut-off value, as follows [19]:

(8) $$N_{ij}\left(r\right)=\frac{4\pi N_{j}}{V}\int_{0}^{r}r^{2}g_{ij}\left(r\right)dr$$

Neutron diffraction is employed to provide insights into the medium-range structural features of the materials. Initially, the partial structure factors $S_{ij}\left(Q\right)$ were computed, and, subsequently, the total structure factors $S_{N}\left(Q\right)$ were derived by combining these partial structure factors, as follows [20,21]:

(9) $$S_{ij}\left(Q\right)=1+4\pi \rho \int_{0}^{R}r^{2}\frac{sin\left(Qr\right)}{Qr}\left[g_{ij}\left(r\right)-1\right]dr$$

(10) $$S_{N}\left(Q\right)={\left(\sum_{i,j=1}^{n}c_{i}c_{j}b_{i}b_{j}\right)}^{-1}\sum_{i,j=1}^{n}c_{i}c_{j}b_{i}b_{j}S_{ij}\left(Q\right)$$

where ρ denotes the mean atomic number density; R represents the maximum value of the integration in real space; $c_{i(j)}$ signifies the fraction of atom i(j); $b_{i(j)}$ represents the neutron scattering length of species i(j).

To discern the oxygen species and clusters characterized by specific polyhedral coordination, we determined the coordination number for each silicon (Si) and aluminum (Al) atom. The cut-off radii for the Si-O and Al-O interactions were determined as the x-axis values corresponding to the first valleys of their respective PDF curves. Consequently, each Al atom was classified as either a network former (four-coordinated Al) or a network modifier. Similarly, each oxygen (O) atom was categorized as bridging oxygen (BO), non-bridging oxygen (NBO), or free oxygen (FO) based on its coordination with the Si and Al atoms. These computations were executed within the Matlab 2019 environment.

## 3. Results

### 3.1. Internal Energy

**Figure 3:**
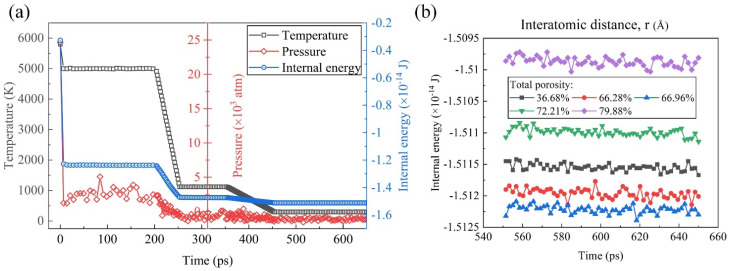
(**a**) Temperature, pressure, and internal energy of the simulated system the during the foaming process. (**b**) Equilibrium internal energy–time curves of samples with different TPs.

Figure 3a illustrates the dynamic behavior of temperature, pressure, and internal energy over time in the molecular dynamic simulations. It was evident from the plot that, as the temperature of the system decreases, both the pressure and internal energy exhibit a corresponding decrease. This observation aligns with fundamental principles of thermodynamics, wherein a decrease in temperature typically results in the reduced kinetic energy of the particles, leading to lower pressure and internal energy within the system. Notably, the internal energy stabilized and remained constant after approximately 500 ps, indicating a transition to equilibrium conditions, where the system reaches a thermodynamically stable state. In Figure 3b, the equilibrium internal energy–time curves are presented, focusing on the last 100 ps of simulation time for systems characterized by different TPs ranging from 36.68% to 79.88%, indicating the robustness of the system under different thermodynamic conditions. The internal energy reflecting the overall stability of the system was in a relatively stable value for each system. With the TP increasing, it decreased firstly and then increased, reaching the minimum when the TP was 66.96%. The observed variation in the internal energy with the changing TP conditions may be attributed to several underlying factors, including alterations in the interatomic interactions, structural rearrangements, and phase transitions induced by variations in the TP.

### 3.2. PDF, CN, and SEM

**Figure 4:**
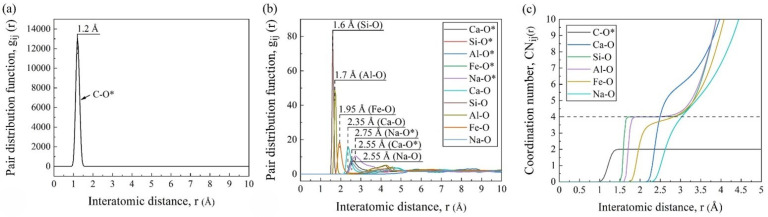
Statistical system information obtained by AAMD simulations: PDF curves (**a**,**b**) and CN curves (**c**) of pairs (O* and O mean the oxygen in CO_2_ and FGCs melt, respectively).

Figure 4a,b shows the PDF curves of different pairs, where the abscissa positions of the first peaks represent the average bond lengths of the pairs. As marked in the figures, the average bond lengths were 1.2 Å for C-O*, 2.35 Å for Ca-O, 1.6 Å for Si-O, 1.7 Å for Al-O, 1.95 Å for Fe-O, and 2.55 Å for Na-O, which were consistent with the other MD simulations [17,22]. It was worth noting that the simulated average bond length of C-O* was longer than that in the CO_2_ molecules (1.16 Å), which was because it was slightly asymmetric with two different C-O bonds (1.28 and 1.31 Å) under the silicate environment [23]. And it can be seen from the PDF curves that the Ca and Na cations had weak and long-distance interactions with the CO_2_ molecules, while the effects of the other cations in silicate were almost negligible.

As shown in Figure 4c, the smooth and wide platform at CN_Si-O_ = 4 confirmed the stable four-coordination structure, i.e., the SiO_4_ tetrahedra. The Al-O pair also had a slightly sloped platform at CN = 4. This was because, in addition to the four-fold coordinated aluminum (AlO_4_ tetrahedra) as the network former, a small amount of Al existed in the other coordination forms as the network modifier [24]. The CN curves of Fe-O, Ca-O, and Na-O gradually increased in inclination, indicating their variable CNs and unstable forms.

**Figure 5:**
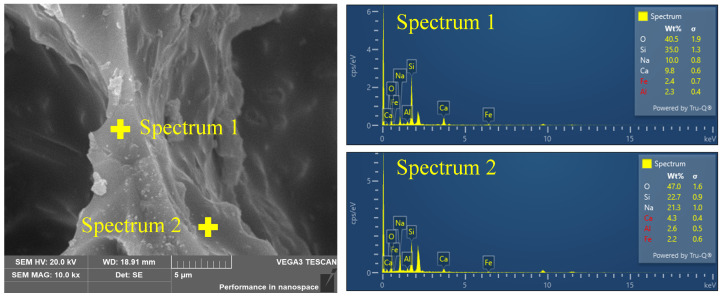
SEM image of FGC accompanied by elemental distributions obtained from energy-dispersive X-ray spectroscopy.

The element content of the FGCs in the pore wall matrix and surface are shown in Figure 5. Notably, a significant disparity in the content of Na cations was observed between the surface and matrix regions, with concentrations of 21.3% and 10.0%, respectively. This difference provides compelling evidence for the interaction between Na cations and CO_2_, suggesting a migration and accumulation of Na cations towards the pore wall surface under the influence of CO_2_. In contrast to the pronounced accumulation of Na cations, the interaction of Ca cations with CO_2_ gas appears to exhibit a different behavior. Despite the acknowledged interaction between Ca cations and CO_2_ molecules, as evidenced by the elemental content analysis, there is no conspicuous accumulation of Ca cations at the pore wall surface. This discrepancy can be attributed to several factors, including the larger ionic radius of Ca compared to Na and the lower mobility of Ca cations within the composite matrix. It is worth noting that the migration of Na cations within the FGC pore wall matrix can have significant implications for the atomic-level structure, particularly concerning the [SiO_4_] tetrahedral units that constitute the backbone of the silicate network. The incorporation of Na cations into the vicinity of the [SiO_4_] units can induce structural modifications through interactions, thereby influencing the connectivity and arrangement of the Si-related structure.

### 3.3. Effects of Foaming Degree on Si-Related Structure

**Figure 6:**
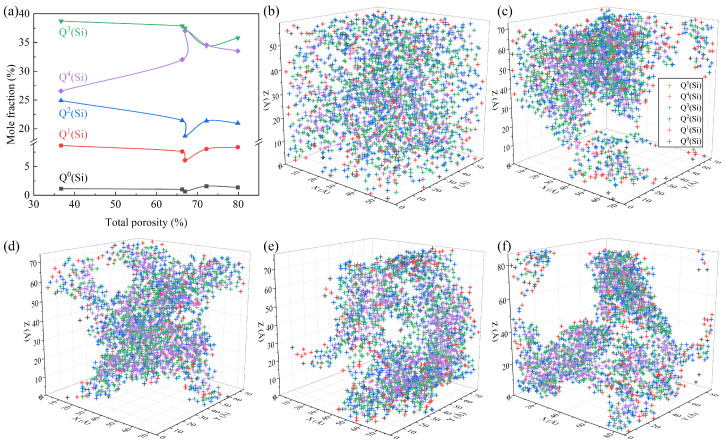
Quantitative Si-related structural information: (**a**) concentration variation in the Q^i^(Si) units; snapshots of the location distribution of different types of Q^i^(Si) units with TPs of 36.68% (**b**), 66.28% (**c**), 66.96% (**d**), 72.21% (**e**), and 79.88% (**f**).

Figure 6 depicts the quantitative Si-related structural information in systems with different TPs. It can be seen from Figure 6a that, in all samples, Q^3^(Si) and Q^4^(Si) corresponding to the groups of [Si_2_O_5_]^2−^ and [SiO_2_] were the dominant types of SiO_4_ units, which was similar to the structural feature of the waste glass [25]. The TP had an obvious impact on the concentration of Q^i^(Si), which could be divided into two stages. During the initial increase in the TP, the proportion of Q^4^(Si) increased at the cost of Q^0^(Si), Q^1^(Si), Q^2^(Si), and Q^3^(Si), and the polymerization degree of the Si-related framework decreased. This is because, with the increase in the TP, the specific surface area of the system increases, and masses of the Si-O_nb_-Me (Me: network-modified cations) linkages are generated and distributed on the pore wall in place of linkages of Si-O_b_-Si, which can reduce the surface tension [26] and thus the surface energy [27]. This is accordance with the changes in the distributions of the Q^i^(Si) species in Figure 6b–d, for which the surface was gradually occupied by Q^i^(Si) with a low BO number. The peak content of Q^4^(Si) occurs at a TP of 66.96%, which is the same as the TP with the lowest internal energy, as shown in Figure 3. With the TP further increasing, the mass fraction of Q^4^(Si) decreased. This may be due to the tendency of inter-pore connectivity with the increasing TP and the breaking of the Si-O bonds caused by the flow of the melt during the pore connectivity process [28,29]. As shown in Figure 6d–f, the network was gradually broken down into small regions by gas molecules and the connected gas channels increased, which explains the formation of macroscopically connected pores. In terms of the Si-related structure, the Q^4^(Si) converted into Q^i^(Si) units with a low degree of polymerization.

### 3.4. Effects of Foaming Degree on Al-Related Structure and Oxygen Species

**Figure 7:**
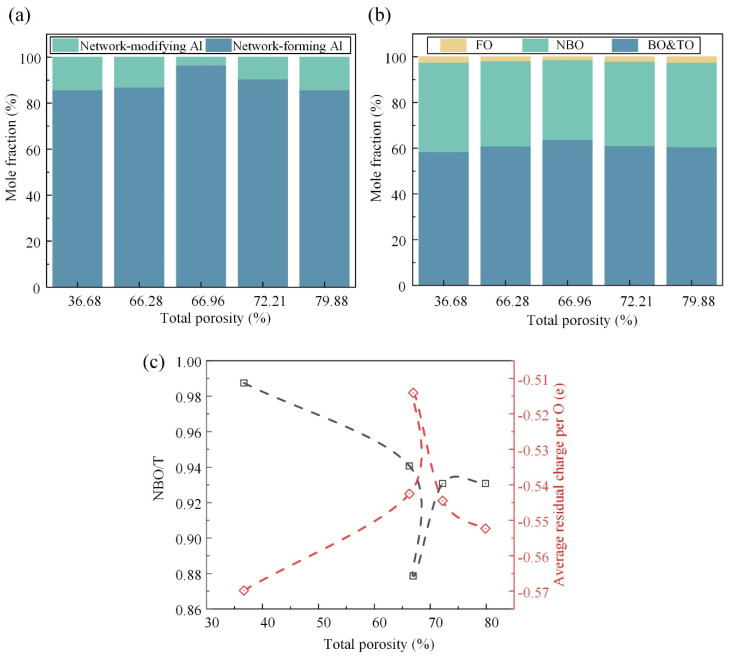
Quantitative information of the Al-related structure and oxygen species and the supporting evidence in the spectra: the concentration variation in the Al-related units (**a**) and oxygen species (**b**) (NBO or O_nb_ means non-bridging oxygen, FO means free oxygen, and TO means tricluster oxygen); (**c**) degree of depolymerization, NBO/T, where T denotes the network-forming Si and Al, and the average residual charge per the O atom.

As shown in Figure 7a, the ratio of the network-forming Al to network-modifying Al first increased and then decreased as a response to the Si-related structural transformation. At the initial stage, the Al cation tended to exist in a stable tetrahedral coordinated form because there was enough compensation charge provided by the Me cations that were removed from the Si-related network. At the later stage, the Al cation acted as a network modifier to balance the charge of the Si-O_nb_ linkage. The oxygen species, as the only anion, could provide additional information about the whole system. Here, the TO was in a very small proportion and was incorporated into the BO. Figure 7b illustrates the intricate relationship between the TP and the oxygen species. It is observed that, as the TP increases, the fraction of the BO initially rises before declining, while the fractions of the NBO and FO exhibit the opposite trend. The BO plays a pivotal role in constructing a continuous, cross-linked network of chemical bonds, contributing to its overall structural integrity and strength. Conversely, the NBO and FO are associated with structural defects, such as breakpoint defects, which compromise the cohesive network of the material. Therefore, the observed variation in the oxygen species fractions suggests a critical transition in the mechanical strength, with the maximum strength achieved at a TP of approximately 66.96%.

Furthermore, Figure 7c provides quantitative metrics for assessing the depolymerization degree and structural stability of the FGC matrix under different TP conditions. The depolymerization degree, quantified by the NBO/T, serves as an indicator of the structural breakdown and weakening within the matrix. A higher NBO/T value corresponds to a greater extent of depolymerization and an increased prevalence of structural weaknesses. Similarly, the average residual charge per oxygen atom serves as a measure of the structural stability, with deviations from zero indicating an unstable structure. It is observed that both the depolymerization degree (NBO/T) and structural stability, as quantified by the residual charge, exhibit turning points at the TP of ~66.96%, aligning with the observed trends in the oxygen species fractions.

**Figure 8:**
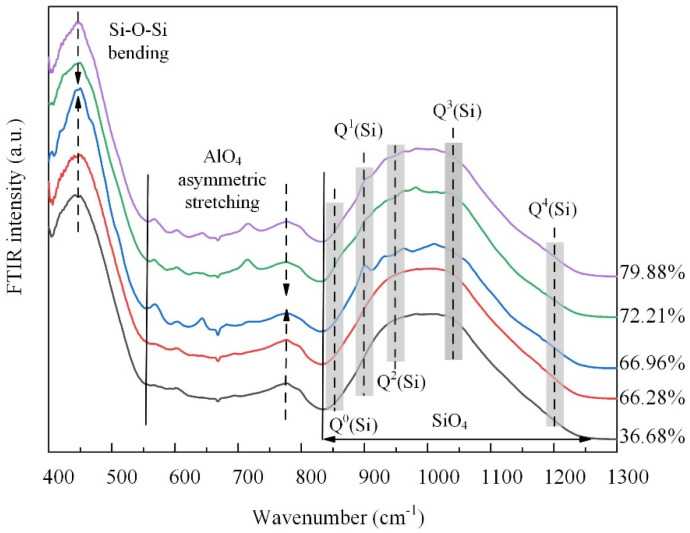
FTIR spectra of the samples.

The structural turning point is corroborated by the FTIR spectral results shown in Figure 8. The low-frequency band from 400 to 600 cm^−1^, attributed to Si-O_b_-Si bending vibrations, and the intermediate frequency band from 600 to 800 cm^−1^, assigned to asymmetric stretching vibrations of AlO_4_ tetrahedra [24], both initially intensified and then weakened. This behavior aligns with the AAMD simulation results and suggests a transitional phase in the structure. That is, the initial increase in the intensity indicates the formation of more regular bonding environments, which subsequently become disrupted as the TP further increases. The high-frequency band from 800 to 1200 cm^−1^ exhibited maximum intensity and was attributed to stretching vibrations in the SiO_4_ tetrahedra [24,30]. For all samples, peaks corresponding to high bridging oxygen were more intense than those for the low bridging oxygen, indicating a higher proportion of network-forming units. Compared to the sample with the 36.68% TP, it is evident that the vibrations of Q^1^(Si), Q^2^(Si), and Q^3^(Si) were weakened, while the vibration of Q^4^(Si) was strengthened as the TP increased. This indicates that, as the TP increased, the silicate network became increasingly disrupted, leading to fewer Q^1^(Si), Q^2^(Si), and Q^3^(Si) species, and a relative increase in Q^4^(Si) species. Except the initial slight enhancement of Q^1^(Si) species, the shift in the intensity centroid in the high-frequency band from higher to lower wavenumbers reflects changes in the Q^i^(Si) units consistent with the simulation results, suggesting initial network ordering followed by structural fragmentation as the TP increases.

**Figure 9:**
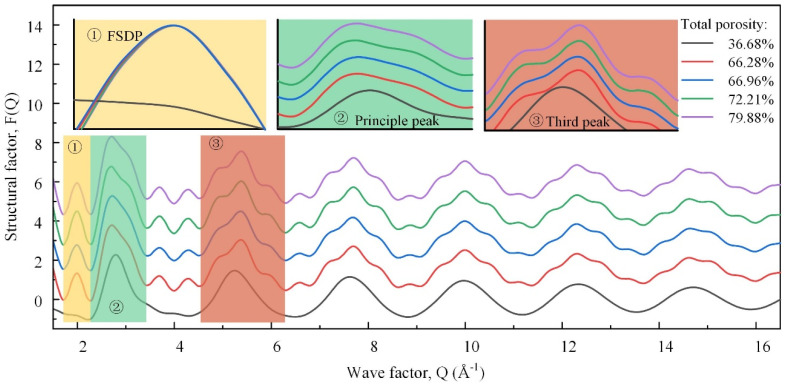
Comparison of samples in total and major partial neutron structure factors.

Figure 9 shows the neutron total structure factors of the samples, which could be associated with different real space length scales. The high-Q (wave factor) oscillations, i.e., the third and higher peaks, were similar but faded away. They were dominated by the degree of order of the nearest-neighbor units in the TO_4_ tetrahedra [31]. The differentiated degree of these peaks was positively correlated with the degree of order, first increasing, and then decreasing. The first sharp diffraction peak (FSDP) was a salient feature that characterized the family of rings in the medium-range order structure [32]. The greater the number of small-sized rings, the higher the wave factor of the FSDP. With the TP increasing, the FSDP shifted to the left first and then to the right, indicating the number of small-sized rings decreased first and then increased. Accordingly, the overall ring size increased first and then decreased, which conformed to the change in the polymerization degree. The second diffraction peak, namely, the principal peak, was related to the size of the tetrahedron [20]. The average size of the tetrahedral building blocks increased as the BO and network-forming Al concentration increased, and the principal peak shifted to the lower-Q peak. Here, the lower-Q and high-Q secondary peaks were enhanced successively, which was attributed to the change in the tetrahedral types.

These neutron diffraction results provide crucial insights into the atomic-scale structural changes underlying the variations in the compressive strength. Initially, the increase in the structural order and polymerization enhanced the mechanical strength, as evidenced by the intensified high-Q peaks and the leftward shift of the FSDP. However, beyond a certain TP threshold, further increases in the TP led to structural fragmentation. This is indicated by the rightward shift and the subsequent decline in the FSDP and principal peak, correlating with a rapid decrease in the compressive strength. These findings highlight the critical balance between maintaining the micro-structural order and managing the macro-porosity to optimize the mechanical properties of foamed glass–ceramics.

The analysis of this study reveals that the structural information of the FGCs exhibits a staged change with the increasing TP. The PDF results indicate that alkali metals in the melt are attracted to the pore walls by the foaming gas (CO_2_), as confirmed in the SEM images. The migration is enhanced with the increase in the foaming gas content, and the migration of alkali metals is increased as the network modifiers affect the structure in the system, while the fusion and collapse of macroscopic pores further impacts the structure. FTIR spectroscopy analysis shows that an initial increase in the TP enhances the ordering of the structure, as manifested by the enhancement of the Si-O_b_-Si and AlO_4_ vibrations, as well as the shift of the Si-O_nb_ stretching vibration, which corresponds to the increase in the compressive strength. This is further confirmed by neutron diffraction, where the high-Q oscillations and the FSDP show an initial enhancement, reflecting an improvement in the short- and medium-range ordering. However, when the TP exceeds a critical threshold, both the FTIR spectroscopy and neutron diffraction data show structural fragmentation: the FTIR bands weaken, the high-Q peaks and FSDP decline, and the main peak shifts, indicating a breakdown in the network connectivity. This structural degradation leads to a rapid decrease in the compressive strength. Thus, the analytical results highlight a critical balance between a moderate TP, which enhances the structural integrity and strength, and an excessive TP, which impairs these properties, underscoring the importance of optimizing the TP for high-performance FGCs.

## 4. Conclusions

This study investigated the short and medium-range structural configurations of foaming glass–ceramics (FGCs) across diverse total porosity levels (from 36.68% to 79.88%). It was found that changes in the total porosity strongly influence local structural features, leading to notable alterations in the compressive strength. The primary conclusions are as follows:

(1) Na cations were observed to migrate towards the surface of the pore walls due to the attraction by CO_2_. The Na content on the surface of pore wall (21.3%) surpassed that within the matrix (10.0%), thereby impacting the polymerization degree.
(2) The quantity of bridging oxygen exhibited an initial rise followed by a decline as the total porosity increased, peaking at 66.96%. The matrix of the pore wall was predominantly occupied by high bridging oxygen, fostering the establishment of a cohesive cross-linked network of chemical bonds, and thereby augmenting the matrix polymerization.
(3) As the total porosity increased, the neutron structure analysis revealed an initial augmentation followed by a subsequent diminution in both the overall ring size and average size of the tetrahedral building blocks. This trend aligned with the observed variation in polymerization degree.
(4) The inflection points of the depolymerization degree and the average residual charge per O atom coincided at a total porosity of approximately 66.96%, which corresponded to the optimum sample. The subsequent increase in the total porosity led to the emergence of interconnected pores and breakpoints within the foaming glass–ceramic, which significantly reduced its compressive strength.

## Figures and Tables

**Table 1:** Chemical compositions (wt.%) of waste glass and incineration bottom ash.

Raw Materials	SiO_2_	Na_2_O	CaO	Al_2_O_3_	MgO	Fe_2_O_3_	K_2_O	SO_3_	Others
Waste glass	63.2	17.7	12.0	1.7	1.8	2.1	0.9	0.2	0.4
Incineration bottom ash	34.8	6.7	10.4	3.9	12.4	24.1	3.4	4.1	0.2

**Table 2:** Potential parameters used in the AAMD simulations.

I	Silicate potential			
	$A_{ij}$ (eV)	$B_{ij}$ (Å)	$C_{ij}$ (eV/Å^6^)	$D_{ij}$ (eV/Å^12^)
Ca^0.945^-O^−0.945^	155,356.043	0.178	42.2587	0
Si^1.89^-O^−0.945^	50,186.0509	0.161	46.2967	0
Mg^0.945^-O^−0.945^	32,587.272	0.178	27.2803	0
Al^1.4175^-O^−0.945^	28,482.1454	0.172	34.577	0
Fe^0.945^-O^−0.945^	8034.05	0.19	0	
Na^0.4725^-O^−0.945^	145,402.3125	0.178	18.8075875	0
O^−0.945^-O^−0.945^	6479.68212	0.276	85.0902	0
II	${\mathrm{CO}}_{2}\;\mathrm{intramolecular}\;\mathrm{potential}\text{:}\;l_{C-o^{*}}=1.162\;Å$ $;\;k_{\theta }=4.6096\;eV/rd$			
III	CO_2_-CO_2_ intermolecular potential			
	ε (eV)	σ (Å)		
C^0.5888^-C^0.5888^	0.002490	2.792		
C^0.5888^-O*^−0.2944^	0.004214	2.896		
O*^−0.2944^-O*^−0.2944^	0.007135	3.000		
IV	More potential between C of CO_2_ and O of silicate			
	$D_{e}$ (eV)	l (Å)	λ (Å)	
C-O	5.0249	1.162	0.2	
V	CO_2_-silicate potential			
	$A_{ij}$ (eV)	$B_{ij}$ (Å)	$C_{ij}$ (eV/Å^6^)	$D_{ij}$ (eV/Å^12^)
O^−0.945^-O*^−0.2944^	3239.84106	0.276	42.5451	0
Ca^0.945^-O*^−0.2944^	155,356.043	0.178	42.2587	0
Si^1.89^-O*^−0.2944^	50,186.0509	0.161	46.2967	0
Mg^0.945^-O*^−0.2944^	32,587.272	0.178	27.2803	0
Al^1.4175^-O*^−0.2944^	28,482.1454	0.172	34.577	0
Fe^0.945^-O*^−0.2944^	8034.05	0.19	0	
Na^0.4725^-O*^−0.2944^	145,402.3125	0.178	18.8075875	0

## Data Availability

The data presented in this study are available on request from the corresponding author.
